# The N400 and Late Positive Complex (LPC) Effects Reflect Controlled Rather than Automatic Mechanisms of Sentence Processing

**DOI:** 10.3390/brainsci2030267

**Published:** 2012-08-14

**Authors:** Jérôme Daltrozzo, Norma Wioland, Boris Kotchoubey

**Affiliations:** 1 CNRS UMR7237, Louis Pasteur University, 12 rue Goethe, Strasbourg F-67000, France; Email: nwioland@free.fr; 2 Institute of Medical Psychology and Behavioral Neurobiology, Eberhard Karls University, Gartenstr. 29, Tübingen D-72074, Germany; Email: boris.kotchoubey@uni-tuebingen.de; 3 CNRS UMR5292, INSERM U1028, Claude Bernard Lyon 1 University, 50 Avenue Tony Garnier, Lyon cedex 01 F-69366, France

**Keywords:** ERP, masking, mask, semantic, priming, control, context, auditory, language, speech

## Abstract

This study compared automatic and controlled cognitive processes that underlie event-related potentials (ERPs) effects during speech perception. Sentences were presented to French native speakers, and the final word could be congruent or incongruent, and presented at one of four levels of degradation (using a modulation with pink noise): no degradation, mild degradation (2 levels), or strong degradation. We assumed that degradation impairs controlled more than automatic processes. The N400 and Late Positive Complex (LPC) effects were defined as the differences between the corresponding wave amplitudes to incongruent words minus congruent words. Under mild degradation, where controlled sentence-level processing could still occur (as indicated by behavioral data), both N400 and LPC effects were delayed and the latter effect was reduced. Under strong degradation, where sentence processing was rather automatic (as indicated by behavioral data), no ERP effect remained. These results suggest that ERP effects elicited in complex contexts, such as sentences, reflect controlled rather than automatic mechanisms of speech processing. These results differ from the results of experiments that used word-pair or word-list paradigms.

## 1. Introduction

The N400 component of event-related potentials (ERPs) is a large negativity with a broad (parietally maximal) scalp distribution, peaking around 400 ms (largest for semantic incongruencies). Among these semantic anomalies, the level of semantic incongruity between a word and a given context is well known to modulate the amplitude of the N400 [1]. The context can be a single word (*i.e.*, in word-pair paradigms, [2]), a sentence [3,4,5] or a full discourse [6,7]. There is extensive literature [8,9,10,11,12,13,14,15,16,17,18,19,20] showing that when the N400 effect (*i.e.*, the N400 to a word within an incongruent context compared to a congruent context) is recorded with a word-pair paradigm, the effect can be elicited without attention to automatic mechanisms [21,22].

When the context is a single word, as in word pair experiments (*i.e.*, a *prime* followed by a *target* word), the context effect is often called *semantic priming.* When semantic priming occurs, the brain activity differs depending on whether the target is semantically related (e.g., *dog*-***cat***) or unrelated (e.g., *dog*-***stone***) to the prime. Semantic priming was first observed in a lexical decision task with higher performance (*i.e.*, higher accuracy and faster responses) to related targets compared to unrelated targets [23]. Importantly, semantic priming can be conscious or unconscious. Indeed, semantic priming data can be explained by controlled predictive processes, controlled integrative processes, or by an automatic mechanism called *Automatic Spreading Activation* (ASA) [24]. According to the ASA model, the mental lexicon is assumed to be a semantic network with related words in neighboring nodes. ASA would occur because the lexical access to a word (e.g., *mountain*) would unconsciously activate the corresponding node [25,26] and spread activation to the neighboring nodes relative to related words (e.g., *summit*). In a predictive mechanism, when a prime is given (e.g., *mountain*) a set of expected words (e.g., *summit*, *hut*, *lake*) is generated and pre-activate the set of corresponding words in the mental lexicon. If the target is among those words, lexical access is facilitated. Other explicit mechanisms are also thought to underlie semantic priming, such as integrative processes. Integrative processes arise when the recognition of a target word (e.g., *summit*) is facilitated because it is judged to be plausible within the semantic context (e.g., *mountain*) [24].

While the contextual effect, referred to as semantic priming, can be driven by an automatic mechanism, such as the ASA, this may not be the case when the target word occurs in a more complex context, such as a full sentence. The mechanisms governing the N400 effect within the context of a sentence has received little attention and the overall output of this research remains unclear. Furthermore, few studies have investigated whether mechanisms governing the N400 effect within the context of a sentence are, by nature, controlled or automatic. Some experiments have used either a degradation technique [27,28,29] or a task manipulation [30,31,32] to examine controlled and automatic processes. A classical assumption is that degradation impairs more controlled than automatic mechanisms [33]. According to McNamara [33] controlled mechanisms should be “reduced or eliminated if primes [corresponding to the sentential context in the present study] were presented outside conscious awareness. Brown and Hagoort […] tested this hypothesis using forward and backward masking of primes in a lexical decision task” ([33], p. 122). Automatic mechanisms are thought to be independent of the level of attention [21,22] and are usually assumed to be activated even without conscious awareness. Therefore, we assumed that under a masking (or degradation) condition, which should reduce the level of consciousness, controlled mechanisms would be more impaired than automatic mechanisms.

Coulson and Brang [29] reported a reduced N400 effect to unmasked (or non-degraded) sentences ending with a masked (or degraded) final target word compared to unmasked sentences ending with an unmasked final word. They concluded that contextual effects of sentences indexed by the N400 reflect both automatic and controlled processes. However, masking (or degrading) only the final word of the sentence may not impair all controlled mechanisms that are thought to affect the processing of the final word [24]. Indeed, controlled predictive mechanisms that unfold during the perception of the sentential context (e.g., [24]) may not be impaired if only the final word of the sentence is masked. Therefore, the reduced N400 effect previously found by these authors [29] in a masked condition may still be generated by controlled sentence-level (predictive) mechanisms but not by automatic mechanisms.

Other studies that degraded the full sentence may have used a too low level of noise. Thus, controlled mechanisms were not reduced to a level where behavioral data show chance level performance. Connolly *et al.* [28] used a test condition with degraded sentences and a control condition without degradation of the sentences. The degradation was done with “informational noise”, *i.e.*, noise built from speech material. Connolly *et al.* [28] built informational noise with 12 superimposed competing voices. Participants were told to perform a semantic categorization task on a visual word displayed after the presentation of the degraded or non-degraded auditory sentence. Under degradation, accuracy was 80%. As performance was well above chance (*i.e.*, 50%), it seems unlikely that the level of degradation was strong enough to impair all controlled mechanisms of speech processing. Therefore, with the assumption that the N400 effect is elicited by controlled sentence-level mechanisms, one would expect to find a remaining N400 effect in this masked condition. Indeed, the authors [28] reported a delayed N400 effect under the condition of degradation. They proposed that the delay found on the N400 effect was due to the increased cognitive load required to process the degraded sentences. Aydelott *et al.* [27] reported a similar study. They recorded the N400 effect to degraded and non-degraded sentences. The N400 effect to degraded sentences was significantly reduced compared to the N400 effect to non-degraded sentences. Unlike Connolly *et al.* [28], they did not use an informational mask but an energetic mask (*i.e.*, artificial noise that does not include speech stimuli). The acoustic degradation consisted of a low-pass filtering of the sentence sound file at 1 kHz. The degradation allowed highly accurate interpretation of the sentences (*i.e.*, performance accuracy of 93%). Thus, the degradation level used by Aydelott *et al.* [27] and the degradation used by Connolly *et al.* [28] were unlikely to impair all controlled sentence-level mechanisms. Therefore, it is possible that the studies of Connolly *et al.* [28] and Aydelott *et al.* [27] used too mild degradations, and, in turn, degradations did not fully impair the controlled mechanisms of sentence-level speech processing. Hence, these studies could not examine whether automatic sentence-level processing mechanisms alone are able to modulate the ERP responses. To examine controlled and automatic mechanisms of speech processing, a stronger degradation (where behavioral data still indicate sentence processing) is required, wherein the controlled mechanisms are impaired to a level where behavioral data indicate that only automatic processing remains. Under strong degradation, the presence of a remaining N400 effect would be evidence that automatic sentence-level mechanisms can modulate the N400. Alternatively, if under strong degradation no N400 effect is found, the conclusion would be that the N400 effect is generated by controlled but not by automatic sentence-level mechanisms.

Using a different approach, Balconi and Pozzoli [30] recorded an N400 effect with and without a semantic judgment task and found this effect to be unaffected by the task. This result was interpreted as reflecting the automaticity of the mechanisms underlying the N400 effect. However, an alternative interpretation would be that the task did not interfere with the controlled sentence-level mechanisms responsible for the N400 effect.

Conversely, the results of Hahne and Friederici [31], and Schön and Besson [32], suggest that the N400 effect obtained with sentences does not reflect automatic mechanisms but exclusively controlled mechanisms. Schön and Besson [32] presented excerpts lasting between 8 s and 20 s from operas (sung *a capella*) under four conditions: The final word of the excerpt was either: (1) semantically congruent with the sentence and sung in tune, or (2) semantically incongruent and sung in tune, or (3) semantically congruent and sung out of tune, or (4) semantically incongruent and sung out of tune. Depending on the instructions, listeners focused their attention on the sentences (*i.e.*, the lyrics) or on the tunes. The authors [32] reported an N400 effect only under the condition where sentences were listened to, but not when the participant listened to the tunes). Similarly, Hahne and Friederici [31], using sentences with syntactic and semantic violations, observed an N400 effect to semantic violation only when participants were asked to listen to semantic violations and ignore syntactic errors. However, it is possible that when participants listened to syntactic violations, that the latest part of the frontal negativity effect could be interpreted as an N400 effect. Thus, it remains unclear whether the attention for the semantic violations was fully abolished under this condition.

In summary, the literature on the automaticity of the N400 effect to sentences remains inconclusive, and motivates the present study.

In response to a word, the N400 is frequently followed by a parietal late positive complex (LPC) peaking around 600 ms after the stimulus. In contrast to the N400 effect, there are no data clearly testing whether an LPC effect to a sentence-level semantic incongruity is due to automatic or controlled mechanisms. The LPC has been thought to reflect semantic integration and conscious understanding [34], confidence in the integration of a word within its context [35], semantic memorization and classification [36,37,38,39], post-decision closure [40], or “repair” of an erroneous sentential structure [41,42]. It is unlikely that all these putative mechanisms are only performed by automatic mechanisms. Rather, the activation of these mechanisms also implies the participation of controlled processes.

In summary, the literature suggests that the occurrence of the N400 effect and the LPC effect within a sentential context may reflect controlled cognitive mechanisms. What remains unclear is whether automatic sentence-level mechanisms can also contribute to these ERP effects.

The aim of the present study is to test the automaticity of sentence-level mechanisms responsible for the N400 effect and the LPC effect through different levels of acoustic degradation.

The experimental design was based on previous ERP experiments of semantic processing, but we manipulated the level of controlled processing differently to take into account the following factors:

(1) Most of these studies were designed under the assumption that automatic and controlled processes are mutually exclusive [22,43]. Yet, a dichotomy between controlled and automatic processes may not exist [14,44,45,46,47,48,49]. Rather, there may be a continuum of processes at different levels of awareness and attention (e.g., [50,51,52]) or on other dimensions as those proposed by Logan [53]. Logan proposed several distinctions of automaticity (speed, effortlessness, autonomy, and lack of conscious awareness) and of non-automaticity (controlled, effortful, or strategic) across different dimensions. In the present study, it was assumed that automatic and controlled processing is differentiated on the basis of the attentional dimension (and conscious awareness). Previous experiments have only used two experimental conditions where the controlled mechanisms were assumed to be either present or absent. In contrast, our design included four experimental conditions of acoustic degradation. In each condition, we expected a different degree of controlled processing corresponding to the degradation level (DL). The extent of controlled processing at each DL was estimated with a *degradation efficiency test* (see the report of the pilot study in the Methods).
(2) Previous studies have only degraded the context of the target word [20]. If only the context is degraded, a backward activation (or *backward priming*, [54]) can occur, *i.e.*, the non-degraded target reactivates the semantic representation of the degraded context. Backward priming is assumed to be a controlled mechanism [33]. Thus, even if the context is strongly degraded, the (controlled) backward priming mechanism would remain and could be wrongly interpreted as an automatic mechanism. The present experiment overcame this confound by degrading the context (*i.e.*, the beginning of the sentence) and the target (*i.e.*, the sentence final word) [20].
(3) In order to avoid the overlapping of a N400 to the target with a P300 due to decision making [55], our experiment did not measure the behavioral performance in response to the target word. Instead, performance was measured to a subsequently presented visual word which appeared after: (i) the auditory sentence had been processed, and (ii) the ERPs of interest recorded. Participants were asked to indicate if the visual word and the final word of the sentence were the same word or different words. Thus, the decision was performed only after the visual word presentation.

It was hypothesized that: (1) Performance on the administered task (word recognition of the final word of the sentence) would decline with degradation, *i.e.*, increased correct response time (RT) and decreased accuracies; (2) The N400 and LPC effects to the final words of the sentences would disappear if, under a degradation condition (where behavioral data still indicate sentence processing), the presence of controlled mechanisms could be ruled out (according to a *degradation efficiency test*, see the report of the pilot study in the Methods).

## 2. Methods

### 2.1. Participants

Twenty right-handed native French-speakers (mean age = 21 years; SD = 2.6; range 18 to 26 years; 10 females) without reported visual, auditory or neurological deficits provided written informed consent for their paid participation. The study was performed as part of a project approved by the ethics committee of the University Hospital of Strasbourg (CCPPRB Alsace No.1), and conformed to the 1964 Declaration of Helsinki.

### 2.2. Auditory Stimuli

The paradigm was built with 100 auditory sentences (duration: 2 to 3 s) presented binaurally to the participants through earphones with sound tubes (ER-2 Etymotic). Peak sound intensity of sentences (without mask) at presentation ranged from 57 to 66 dB-A according to a sound level meter (Voltcraft 329 Conrad Electronic, Inc.).

Fifty sentences ended with a semantically congruent target word, and the other 50 sentences ended with an incongruent word. Congruent and incongruent sentences were presented in a pseudo-random order. The congruent sentences were selected from the corpus of a thesis of phonetics [56]. The cloze probability of a target word (*i.e.*, the percentage of participants who spontaneously complete the sentence with this word, see [2]) was based on the responses of 200 participants. All congruent sentences had a cloze probability higher than 20% (*M* = 47.9%; SD = 21.3%; range: 21%–93%). We assumed that by increasing target homogeneity, recognition time would be more homogeneous as well. Thus, we selected only disyllabic targets. In addition, we expected to obtain a more homogeneous recognition time and, hence, more homogeneous ERP waveforms if all the targets started by a consonant in a CV, CCV, CVC or CVCC arrangement (“C” = consonant; “V” = vowel). The initial consonants /f/, /s/, /ò/, /l/ and /R/, being rather short or long in French, were avoided. All target words were nouns. Auditory incongruent and congruent targets were matched for lexical frequency (occurrence in millions from Lexique 3.45; [57]): means (SD) 25 (63) and 54 (86) (*t* = 1.47, *p* > 0.05), number of letters: 6.4 (1.1) and 6.5 (1.3) (*t* = 0.23, *p* > 0.05), and duration: 528 (96) ms and 531 (85) ms (*t* = 0.16, *p* > 0.05), respectively.

To segment the acoustic signal of the sentential context (*i.e.*, the sentence without the final word) from the acoustic signal of the target (*i.e.*, the sentence final word), the target onset was estimated using visual and auditory cues. The visual cue was based on the time-frequency display of the acoustic signal (Adobe Audition 1.5). Listening separately to the sentential context and the target provided an auditory cue which further confirmed accuracy of the acoustic segmentation based on the visual cue.

The 50 incongruent sentences were built from the 50 congruent sentences by using the same truncated sentences (*i.e.*, the sentence truncated from the final target word) followed by an incongruent target word. All words of the sentences including the final word were presented at the natural speech speed (*i.e.*, they were played as they were recorded). Thus, there was no additional inter-stimulus interval between the penultimate word and the final word of the sentence. For examples of the material, see the Appendix.

The full sentences (*i.e.*, the context and the target) were acoustically degraded. This degradation was performed by modulating the acoustic signal [58] with a pink noise using Adobe Audition 1.5. Unlike the white noise (used for audiometric tests, e.g., [59,60]), the pink noise sounds more like a noise of the natural environment because the spectrum of the pink noise compensates for the ear sensitivity (lower in low than in high frequencies).

**Figure 1:**
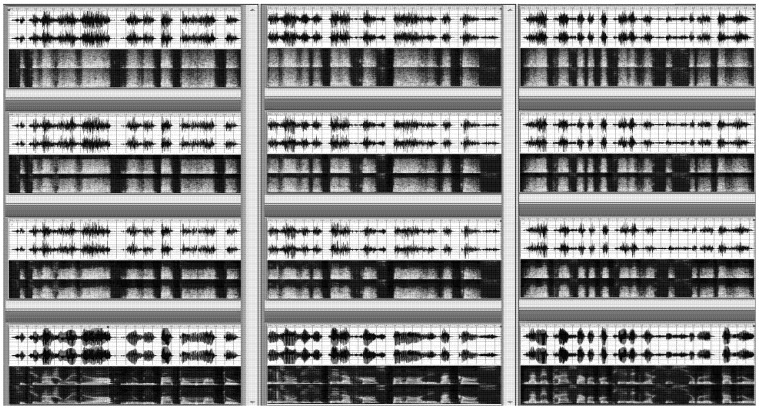
Waveforms and spectrograms of three sentences on the left (*Si tu vas jouer dehors*, *n’oublie pas ton manteau.*), middle (*Ils ont visité la France pendant les vacances.*), and right panel (*La maîtresse a recopié l’exercice sur le tableau.*) in the four degradation conditions. The bottom panel shows the no degradation condition (DL0), the second panel from the bottom shows the “low” degradation condition (DL1), the second panel from the top shows the “medium” degradation condition (DL2), and the top panel shows the “strong” degradation condition (DL3). Waveforms vertical scale range from 0 to −∞ (Unit: dB). Spectrogram vertical scale range from 0 kHz to 5 kHz (linear scale). Waveform and spectrogram horizontal time axis range from 0 s to the sentence duration, *i.e.*, 2.08 s for the left panel, 2.57 s for the middle panel, and 2.70 s for the right panel.

Four degradation levels (DLs) were used: no degradation (DL0), “low” degradation (DL1), “medium” degradation (DL2) and “strong” degradation (DL3). DL3 was obtained by: (1) a modulation of the sentences acoustic signal with a pink noise (intensity: −10.8 dB generated and measured by Adobe Audition 1.5) and (2) an amplification of the degraded signal (according to the root mean squared overall intensity computed by Adobe Audition 1.5). The resulting signal to noise ratio ranged from −2.85 to 0.08 dB (as measured by Adobe Audition 1.5). DL2 and DL1 were obtained with the same procedure except that before modulation, the pink noise was low-pass filtered using a fast Fourier transformation (with Adobe Audition 1.5) at 4000 Hz and 2000 Hz, respectively. This procedure resulted in filtered pink noise of −11.4 dB and −11.8 dB, respectively, and after modulation, resulted in a signal to noise ratio ranging from −3.09 to −0.16 dB for DL2 and from −3.24 to −0.30 dB for DL1 (see Figure 1).

The four degradation conditions were presented in a blocked design in the following order: first DL3, then DL2, DL1, and finally DL0. In each block, the same 100 sentences (50 congruent and 50 incongruent sentences pseudo-randomly mixed) were presented. We applied this experimental design, rather than a mix of varying levels of degradation within the same block of trials because this allowed us to present the same stimuli at several (>2) DL (given the complexity of a sentence, using different sentences for testing the same condition would have introduced some noise because different sentences could hardly be matched with sufficient precision on all relevant parameters) without a strong learning effect and with the same group of participants (using a between-groups design would introduce between-group variation) (see also the last section of the Discussion).

A pilot study referred to as *degradation efficiency test* (see next section) estimated the degree to which sentential processing was impaired by acoustic degradation with a semantic judgment task. The aim of this pilot study was to estimate the contribution of controlled processes for speech processing at each DL.

#### 2.2.1. Degradation Efficiency Test

The aim of this pilot study was to test how the acoustic degradations at DL1, DL2, and DL3 impaired the ability to discriminate between congruent and incongruent sentences. This discrimination was compared to a control condition where sentences were not degraded (DL0). The *degradation efficiency test* was performed by participants who did not participate in the primary (ERP) study. It was assumed that, if, at a given degradation level, the overall accuracy in the semantic judgment task (*i.e.*, discrimination between a congruent and an incongruent sentence) was not significantly different from chance, while performances nevertheless differed between congruent and incongruent sentences, then the mechanisms responsible for this behavioral difference would be automatic rather than controlled.

#### 2.2.2. Methods of the Degradation Efficiency Test

Eleven right-handed native French-speakers (age mean = 22 years, SD = 2.2, range 19–25 years, 6 females) without self-reported visual, auditory, or neurological deficits participated and provided a written informed consent. They did not participate in the primary study.

Participants were presented the same sentences as in the primary study, with the same list of sentences (hence the same number of trials) and with the same block order. Unlike the primary study, sentences were not followed by a visual word presentation (see next section). Instead, a visual probe followed the sentence presentation with an inter-stimulus interval of 1.5 s. One probe displayed the letters “I” (for incongruent sentences) and “C” (for congruent sentences) on the left and right side of the screen, respectively; the other probe presented “C” and “I” on the right and left side of the screen, respectively. If the letter “I” was presented on the left side of the screen, participants had to press the left mouse button if they judged the sentence to be incongruent and the right button otherwise. If the letter “C” was presented on the left side of the screen, they had to press the left button for congruent sentences and the right button otherwise. The presentation of each probe was counterbalanced between trials and the probability of each probe display was 50%. The participants were asked to respond as quickly and accurately as possible, and to make a guess if necessary. Two seconds after the participant’s response, the next sentence was presented.

Accuracies and RT for correct responses were analyzed using repeated-measures analyzes of variance (ANOVAs) with Tukey *post hoc* tests, and with Degradation Level (DL: 4 levels), and semantic congruency (congruent, incongruent) within-participant factors. All these tests were conducted using Statistica version 6. Accuracy was tested for significance against chance expectation (*i.e.*, 50%) with a Bonferroni corrected binomial test. Greenhouse-Geisser correction was applied when applicable [61].

#### 2.2.3. Results of the Degradation Efficiency Test

The data are presented in Table 1. Accuracy was collapsed across experimental conditions, that is, whether the target sentence final word was semantically congruent or incongruent with the sentence. Accuracy decreased with increasing degradation (*F*(3,30) = 125, *p* < 0.001), and was significantly greater than the chance level of 50% (all *ps* < 0.001) except at DL3. *Post hoc* tests indicated that accuracy at DL3 was lower than accuracies at other levels (all *ps* < 0.001). Accuracy at DL2 was lower than accuracy at DL1 and DL0 (all *ps* < 0.001) and lower at DL1 than at DL0 (*p* = 0.046).

RT increased with increasing degradation (*F*(3,30) = 9.50, *p* = 0.001). *Post hoc* tests indicated that RT was greater at DL3 than at any other level (*ps* < 0.001), greater at DL2 than at DL1 and DL0 (*ps* < 0.001), and greater at DL1 than at DL0 (*p* = 0.020). *Post hoc* tests indicated that RT was greater at DL3 than at DL1 and DL0 (all *ps* < 0.05), and greater at DL2 than at DL0 (*p* = 0.03).

Participants made more semantic judgment errors to incongruent target words than to congruent targets, as shown by a semantic congruency effect (main effect of congruency: *F*(1,10) = 16.2, *p* = 0.002) that did not vary significantly across DLs (DL by congruency interaction: *F*(3,30) = 1.06, *p* > 0.05).

The semantic congruency effect was also found with RT: participants responding faster to congruent targets than to incongruent targets (main effect of congruency: *F*(1,10) = 5.88, *p* = 0.036). This difference did not vary significantly across DL (DL by congruency interaction: *F*(3,30) = 3.05, *p* > 0.05).

In summary, accuracy and RT for discriminating congruent and incongruent sentences was better when the sentence was congruent than when the sentence was incongruent at all DLs, including at DL3, where these semantic congruency effects were the largest as compared to the effects at other DLs (Table 1).

**Table 1:** Behavioral data from the Degradation Efficiency Test. Accuracy (%) and correct response time (ms) for each degradation level (no degradation: DL0; “low” degradation: DL1; “medium” degradation: DL2; “strong” degradation: DL3) and for the two conditions: (1) when the sentence ends with a semantically congruent target or (2) an incongruent target. The performance collapsed across all experimental conditions is reported on the left side of the Table. *M* = Mean across participants, *SEM* = Standard error of the mean, all *p*-values are tests against chance performance (*i.e.*, 50%) with a Bonferroni corrected binomial test, *n.s.* = non significant (Bonferroni corrected significance threshold: 0.006).

	Semantic Congruency Effects								
	Accuracy (%)								
	All			Congruent			Incongruent		
	*M*	*SEM*	*p*	*M*	*SEM*	*p*	*M*	*SEM*	*p*
DL3	57.9	1.9	*n.s.*	62.0	2.8	*n.s.*	53.9	3.4	*n.s.*
DL2	85.7	2.5	*	89.9	1.9	*	81.6	3.6	*
DL1	94.9	1.1	*	95.9	1.2	*	93.9	1.6	*
DL0	98.1	0.5	*	98.3	0.5	*	98.0	0.6	*
	**Correct Response Time (ms)**								
DL3	892	126		864	109		920	144	
DL2	754	113		684	82		821	145	
DL1	659	95		617	72		699	119	
DL0	563	45		558	42		568	48	

#### 2.2.4. Discussion of the Degradation Efficiency Test

Accuracy for discriminating congruent and incongruent sentences at DL3 was at chance level. Accuracy nevertheless differed between congruent and incongruent sentences. These data suggest that participants semantically processed sentences with automatic rather than controlled sentence-level mechanisms. Here, we assume that any controlled mechanisms required to perform the task would, if activated, induce a deviation from chance. We also assume that automatic mechanisms (that may or may not occur with the task, *i.e.*, that are more task independent than controlled mechanisms), may or may not induce a deviation from chance.

At other DLs than DL3, accuracies deviated from chance. Therefore, the sentential congruency effects could result from automatic or controlled sentence-level mechanisms. Thus, at DL0, DL1, and DL2, the activation of controlled sentence-level mechanisms cannot be excluded.

Using pink noise, individual words of the sentences may have been more degraded than others, hence, may have been processed at a controlled level. Thus, even though the chance-level performance at DL3 suggested that controlled sentence-level mechanisms were unlikely, other controlled mechanisms at the single word-level may remain at DL3. If such controlled mechanisms had an effect on data at DL3 (e.g., individual words of the congruent sentences being more semantically congruent with the target final word than individual words of the incongruent sentences), we would expect to find a N400 effect at DL3 in the primary study according to the literature (see Introduction of the primary study). However, the ERP responses at DL3 do not show a trend for an N400 effect (see Results of the primary study).

The lack of an N400 effect at DL3 further indicated that even automatic mechanisms at the single word-level that are known to elicit an N400 effect (see Introduction of the primary study) were negligible with our sentence material.

In summary, we may conclude that, at DL3, congruent and incongruent sentences are most probably discriminated through automatic sentence-level mechanisms. Thus, controlled sentence-level mechanisms and (automatic and controlled) single word-level mechanisms would exert only a minor effect on this discrimination. Furthermore, at DL0, DL1, and DL2, the activation of controlled sentence-level mechanisms cannot be excluded.

### 2.3. Visual Stimuli

**Figure 2:**
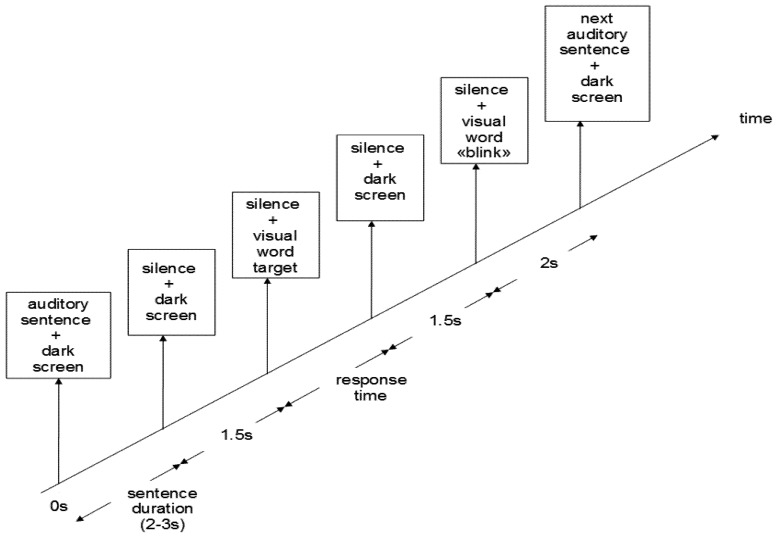
Sequence of stimulus presentation.

For the primary (ERP) study, behavioral data were recorded with a recognition task. To record performance data, to control the level of attention to the final word of the auditory sentence (*i.e.*, the “ERP target word”), and to check that the sentences were semantically processed, a visual (“recognition target”) word was presented after each auditory sentence with an inter-stimulus interval of 1.5 s (*i.e.*, after the ERPs to the ERP target had been recorded, see Figure 2). The visual word was, on average, 10 cm long and 1.5 cm high and was presented at a distance of about 70 cm (with a vertical viewing angle of 1.2° and a mean horizontal angle of 8.1°). The visual word was displayed in white lower case on a dark background in the center of a 13-inch computer screen. Participants were asked to fixate the center of the screen where the probe was displayed during the whole test.

Two types of visual recognition target words were presented randomly with equal probability: “repeated” visual words (*i.e.*, words that were identical to the ERP target word) and “new” visual words (*i.e.*, words that differed from the ERP target word). Since, repeated visual words were identical to the auditory targets, half of the repeated visual words were semantically congruent with the congruent sentences and half of the repeated visual words were semantically incongruent with the incongruent sentences.

To test whether the sentences were semantically processed in all conditions of degradation, we checked that the semantic congruency of the sentential context with the visual word improved the visual word recognition performance. This test was performed by crossing the sentential Congruency factor with the visual target Repetition factor using new visual words as follows: half of the new words were semantically congruent with the sentence and the other half were incongruent with the sentence. Thus, each block (first block at DL3, second block at DL2, third block at DL1, and last block at DL0) of 100 sentences included: 25 congruent sentences presented with a repeated visual word (which was congruent with the sentence), 25 congruent sentences presented with a new visual word (which was incongruent with the sentence), 25 incongruent sentences presented with a repeated visual word (which was incongruent with the sentence), and 25 incongruent sentences presented with a new visual word (which was congruent with the sentence). The order of presentation of the pair of stimuli (auditory sentence and visual word) within the list of 100 trials was pseudo-randomized across blocks and participants. Incongruent and congruent visual words were matched for lexical frequency (occurrence in millions from Lexique 3.45; [57]): means (SD) 24 (35) and 77 (137) (*t* = 1.25, *p* > 0.05), number of letters: 7.1 (1.4) and 6.3 (1.3) (*t* = 1.72, *p* > 0.05), and duration: 528 (96) ms and 531 (85) ms (*t* = 0.16, *p* > 0.05), respectively. All visual words were disyllabic. Words were displayed on a computer screen until a response was recorded.

### 2.4. Procedure

Participants were told to listen carefully to the auditory sentences and to perform a recognition task on the final word of the sentence. The forced-choice recognition task was based on Deacon *et al.* [62]. Participants were to press the left or right mouse button depending on whether the visual word (presented after the auditory sentence) was identical (“repeated” word) or not (“new” word) to the target word (the final word of the auditory sentence). The association between hand side (left or right) and response (“repeated” or “new” word) was balanced across participants. Participants were instructed to respond as fast and as accurately as possible, and to only guess if necessary. After the mouse button was pressed, and after 1.5 s, the word “blink” was presented visually. Participants were instructed that they could blink during this presentation and should avoid blinking at other times [12]. The message stayed on the screen for 1.5 s and was followed by a dark screen lasting for 2 s. A new auditory sentence was then presented (Figure 2).

### 2.5. ERP Data Acquisition and Quantification

The electroencephalography (EEG) was recorded with Ag-Ag/Cl electrodes placed according to the international 10–20 system on the following sites: Fz, Cz, Pz, P3, P4, C3, C4, F3, F4. The reference was taken at the nose and the ground at a prefrontal midline site. The impedance was kept under 10 kΩ. The electro-oculogram (EOG) was recorded with two pairs of electrodes, supra- and infra-orbitally at the right eye (vertical EOG) as well as from the left and right orbital rim (horizontal EOG). The EEG and EOG were acquired on a Neuroscan unit with band-pass filtering (0.1 to 70 Hz) and 500 Hz sampling. ERP data were obtained by averaging EEG epochs, *i.e.*, the EEG around each stimulus onset: 100 ms pre-stimulus onset and 1500 ms post-stimulus onset. All EEG epochs were corrected for blinks and eye movements with the Gratton *et al.* [63] method using the EOG. This procedure uses individual EOG and EEG trials recorded during the experimental session to estimate a propagation factor that describes the relationship between the EOG and the EEG. This factor is used to estimate (from the EOG signal) the EOG noise spread to the EEG. This noise is then subtracted from the EEG. After this procedure, a baseline correction was applied using the prestimulus data. Finally, EEG epochs containing an absolute voltage larger than 70 µV were considered as outliers and were rejected from the analysis. On average, the number of remaining trials per participant was 48 (range: 41 to 50) for congruent targets and 49 (range: 42 to 50) for incongruent targets.

A first analysis was performed without an *a priori* choice of time intervals of the N400 effect and LPC effect across DLs. The mean electric potential amplitudes in 50 ms consecutive time windows were analyzed. Because of the increased likelihood of type I errors associated with the large number of comparisons, only effects that reached significance in at least two consecutive time windows were considered significant [64]. The behavioral and ERP data were analyzed using a repeated-measures ANOVA with Tukey *post hoc* tests. Behavioral data were analyzed with DL (4 levels), Target repetition (repeated, new) and semantic congruency (congruent, incongruent) within-participant factors. To test the distribution of the ERP effects, three regions of interest were selected as levels of a topographic within-participant anteroposterior factor: frontal (F3, FZ, F4), central (C3, CZ, C4), and parietal (P3, PZ, P4) regions and three regions of interest as levels of a laterality factor: left (F3, C3, P3), midline (FZ, CZ, PZ), and right (F4, C4, P4) regions. The Greenhouse-Geisser correction was applied when applicable [61]. All these tests were applied with Cleave and Statistica version 6. Accuracy was tested for significance against chance expectation (*i.e.*, 50%) with a Bonferroni corrected binomial test.

**Figure 3:**
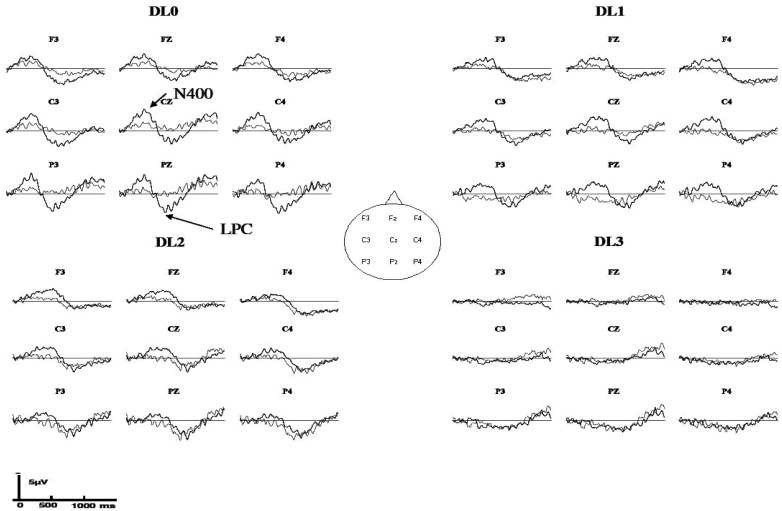
Grand averaged event-related potentials (ERPs) to incongruent targets (thick line) and congruent targets (thin line) at each degradation level (no degradation: DL0; “low” degradation: DL1; “medium” degradation: DL2; “strong” degradation: DL3) (*N* = 20 participants, vertical unit: µV with negativity upward, horizontal unit: ms).

**Figure 4:**
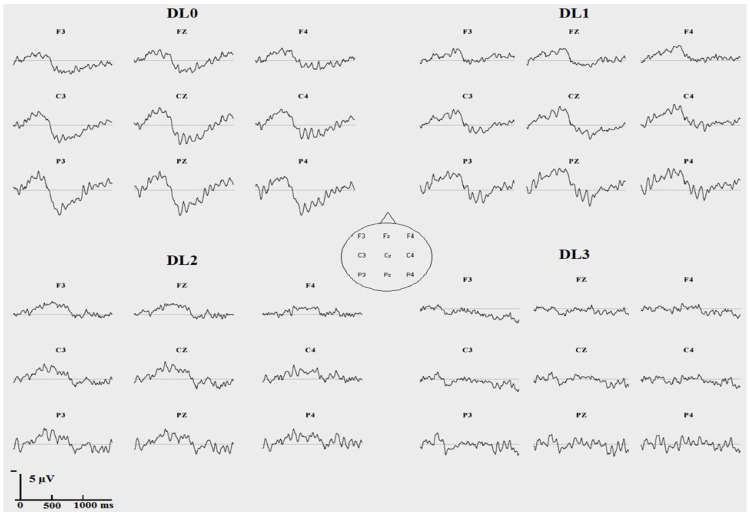
Grand averaged subtraction waveforms between ERP to incongruent targets and ERP to congruent targets at each degradation level (no degradation: DL0; “low” degradation: DL1; “medium” degradation: DL2; “strong” degradation: DL3) (*N* = 20 participants, vertical unit: µV with negativity upward, horizontal unit: ms).

A second analysis was performed with an *a priori* choice of time intervals of the N400 effect and LPC effect across DLs based on the grand-averaged ERPs (Figure 3 and Figure 4). Repeated-measures ANOVA with Tukey *post hoc* tests were performed for each DL and for each ERP effect with an *a priori* time window using the same factors as in the previous analysis except that the DL factor was not included.

A third analysis was performed to estimate the latency of the congruency effects without an *a priori* choice of time intervals. The mean electric potential amplitudes in 50 ms consecutive time windows were analyzed for each DL. Repeated-measures ANOVA with Tukey *post hoc* tests were performed using the same factors as in the second analysis. Because of the increased likelihood of type I errors associated with the large number of comparisons, only effects that reached significance in at least two consecutive time windows were considered significant [64].

### 3.1. Behavioral Results

These data are presented in Table 2. As expected, accuracy (collapsed across experimental conditions, *i.e.*, whether the visual word was repeated or new, semantically congruent or incongruent with the sentence) decreased with increasing degradation (*F*(3,57) = 164, *p* < 0.001) remaining different from the chance level of 50% (all *ps* < 0.001). *Post hoc* tests indicated that accuracy at DL3 was lower than accuracies at other levels (all *ps* < 0.001). Accuracy at DL2, DL1, and DL0 did not differ significantly (all *ps* > 0.05). RT to correct responses increased with increasing degradation (*F*(3,57) = 96.4, *p* < 0.001). *Post hoc* tests indicated that the RT was greater at DL3 than at any other level (*p* < 0.001), greater at DL2 than at DL1 and DL0 (*p* < 0.001), and greater at DL1 than at DL0 (*p* = 0.020).

**Table 2:** Behavioral Data of the primary Event-Related Potential (ERP) Study. Accuracy (%) and RT for correct responses (ms) for each degradation level (no degradation: DL0; “low” degradation: DL1; “medium” degradation: DL2; “strong” degradation: DL3) and for the four conditions: (1) when the visual word presented after the sentence is the same as the last word of the auditory sentence; or (2) a new word; and (3) when this visual word is semantically congruent with the sentence or (4) incongruent. The performance collapsed across all experimental conditions is reported on the left side of the Table. *M* = Mean across participants, *SEM* = Standard error of the mean.

			Repetition Effect				Semantic Congruency Effect			
	Accuracy (%)									
	All		Repeated		New		Congruent		Incongruent	
	*M*	*SEM*	*M*	*SEM*	*M*	*SEM*	*M*	*SEM*	*M*	*SEM*
DL3	77.1	1.5	67.3	3.5	87	1.8	81.2	2.1	73.2	1.4
DL2	93.4	0.5	87.7	0.9	99.2	0.3	97.7	0.6	89.3	0.7
DL1	95.2	0.4	91.5	0.7	98.9	0.3	98.6	0.4	91.9	0.5
DL0	96.5	0.3	94.3	0.5	98.7	0.3	98.4	0.4	94.6	0.4
	**Correct Response Time (ms)**									
DL3	1037	53	1001	47	1072	61	1005	46	1069	61
DL2	757	42	737	42	776	42	689	37	825	47
DL1	653	45	596	36	709	54	629	41	677	49
DL0	586	43	548	45	624	41	592	40	580	45

The participants made more recognition errors to repeated words (misperceived as new) than to new words (misperceived as repeated) as shown by a repetition effect that increased with the DL (target repetition by DL interaction: *F*(3,57) = 8.61, *p* = 0.007). *Post hoc* tests showed a significant target repetition effect at DL2 and DL3 only (*p* < 0.001). RT decreased with increasing degradation (target repetition by DL interaction: *F*(3,57) = 8.26, *p* < 0.001). *Post hoc* tests indicated that the target repetition effect with RT was significant at DL0 and DL1 (*p* < 0.001).

Accuracy differences between congruent and incongruent visual words increased with the DL (congruency by DL interaction: *F*(3,57) = 3.89, *p* = 0.044). *Post hoc* tests showed a significant congruency effect at each DL (DL0: *p* = 0.042, DL1, DL2, and DL3: *p* < 0.001). The semantic congruency effect on visual words recognition was also found with RT. Smaller RT to congruent words than to incongruent words varied across DL (congruency by DL interaction: *F*(3,57) = 36.6, *p* < 0.001). *Post hoc* tests showed a significant effect at DL1 and DL2 (*p* < 0.001), and at DL3 (*p* = 0.013), but not at DL0.

In summary, as expected, overall accuracy decreased and RT increased with more degradation. Target recognition was found at each degradation as indicated by a repetition effect at DL0 and DL1 (with the RT) and at DL2 and DL3 (with accuracy). Sentences were processed at each DL as indicated by a semantic congruency effect on visual word recognition at DL0 (with accuracy) and at DL1, DL2, and DL3 (with accuracy and the RT).

### 3.2. ERP Results

Except in the strong degradation condition (DL3), the grand averaged ERPs to auditory word targets showed different ERP waveforms when the target was presented within a congruent or an incongruent sentential context (Figure 3 and Figure 4). The grand-averaged ERPs suggested a larger N400 (and possibly N2, see Discussion) to incongruent than to congruent targets at DL0 (between 100 ms and 500 ms), at DL1 (between 200 ms and 600 ms), and at DL2 (between 250 ms and 800 ms) but no effect at DL3. Following the N400 effect, the grand-averaged ERPs suggested also a larger LPC to incongruent than to congruent targets at DL0 (between 500 ms and 1200 ms), at DL1 (between 600 ms and 1200 ms), and possibly at DL2 (between 800 ms and 1300 ms) but no effect at DL3.

#### 3.2.1. Statistical Analysis without *a Priori* Time Window of Analysis

ERP effects were tested statistically with a repeated-measures ANOVA with DL (4 levels), semantic congruency (2 levels), anteroposterior (frontal, central, parietal), and laterality (left, midline, right) as within-participant factors and were computed using 50 ms windows (Table 3). The main effect of congruency was significant between 100 and 500 ms (*F*(1,19) = 16.6, *p* < 0.001) and 850 ms to 1000 ms (*F*(1,19) = 6.16, *p* = 0.023). Congruency significantly interacted with laterality in the 800 to 900 ms latency range (*F*(2,38) = 6.17, *p* = 0.005). *Post hoc* comparisons indicated that this was due to a left and midline distribution of the congruency effect (left region: *M* = 0.789 µV, *p* < 0.001; midline: *M* = 0.878 µV, *p* < 0.001; right: *M* = 0.432 µV, *p* > 0.05). Congruency did not significantly interact with DL between 100 and 500 ms (*F*(3,57) = 0.12, *p* = 0.950).

Congruency interacted with DL between 900 and 1000 ms (*F*(3,57) = 4.45, *p* = 0.012), indicating that the congruency effect was significant only at DL1 (*M* = 2.01 µV, *p* = 0.025) but not at DL0 (*M* = 0.05 µV, *p* > 0.05), DL2 (*M* = 0.01 µV, *p* > 0.05), and DL3 (*M* = 1.17 µV, *p* > 0.05). Other interactions with the congruency factor were not significant.

**Table 3:** ERP Semantic Congruency Effects at Each Degradation Level. Congr = Congruency; Congr × DL = Congruency × Degradation Level interaction; Congr × Lat = Congruency × Laterality interaction; Statistical significance threshold: 0.01 (**) or 0.05 (*).

windows (ms)	Congr	Congr × DL	Congr × Lat
0–50			
50–100			
100–150	**		
150–200	**		
200–250	**		
250–300	**		
300–350	**		
350–400	*		
400–450	**		
450–500	**		
500–550			
550–600			
600–650			
650–700			
700–750			
750–800			
800–850			**
850–900	*		*
900–950	*	*	
950–1000	*	*	
1000–1050			
1050–1100			
1100–1150			
1150–1200			
1200–1250			
1250–1300			
1300–1350			
1350–1450			
1450–1500			

#### 3.2.2. Statistical Analysis with an *a Priori* Time Window of Analysis

ERP effects were tested statistically with a repeated measures ANOVA with semantic congruency (2 levels), anteroposterior (frontal, central, parietal), and laterality (left, midline, right) as within-participant factors and were computed for each DL and each ERP effect using *a priori* time windows based on a visual inspection of the grand-averaged ERPs (Figure 3 and Figure 4).

At DL0, we tested the effect of congruency between 100 ms and 500 ms, showing an N400 effect (and possibly N2 effect, see Discussion) with a main effect of congruency (*F*(1,19) = 7.37, *p* = 0.014) (without significant interaction with the congruency).

We also tested the effect of congruency between 500 ms and 1200 ms, showing a LPC effect with a main effect of congruency (*F*(1,19) = 14.28, *p* = 0.001). Congruency interacted with Anteroposterior (*F*(2,38) = 8.70, *p* = 0.004). *Post hoc* tests indicated that the congruency by anteroposterior interaction was due to a LPC effect at frontal (*M* = 1.84 µV), central (*M* = 2.00 µV), and parietal sites (*M* = 2.38 µV) (all *ps* < 0.001). Congruency interacted also with laterality (*F*(2,38) = 6.15, *p* = 0.005). *Post hoc* tests indicated that the congruency by laterality interaction was due to a LPC effect at left (*M* = 1.14 µV), midline (*M* = 2.14 µV), and right sites (*M* = 1.55 µV) (all *ps* < 0.001).

At DL1, we tested the effect of congruency between 200 ms and 600 ms, showing an N400 effect (and possibly N2 effect, see Discussion) with a main effect of congruency (*F*(1,19) = 13.22, *p* = 0.002). Congruency interacted with laterality (*F*(2,38) = 6.70, *p* = 0.005). *Post hoc* tests indicated that the congruency by laterality interaction was due to an N400 effect at left (*M* = −0.96 µV), midline (*M* = −1.42 µV), and right sites (*M* = −1.60 µV) (all *ps* < 0.001).

We also tested the effect of congruency between 600 ms and 1200 ms, showing a LPC effect with an interaction between congruency and anteroposterior (*F*(2,38) = 6.53, *p* = 0.011). *Post hoc* tests indicated that the congruency by anteroposterior interaction was due to a LPC effect at central (*M* = 0.70 µV, *p* = 0.001) and parietal sites (*M* = 1.29 µV, *p* < 0.001). Congruency interacted also with laterality (*F*(2,38) = 8.32, *p* = 0.002). *Post hoc* tests indicated that the congruency by laterality interaction was due to a LPC effect at left (*M* = 0.75 µV) and midline sites (*M* = 1.00 µV) (all *ps* < 0.001).

At DL2, we tested the effect of congruency between 250 ms and 800 ms, showing an N400 effect (and possibly N2 effect, see Discussion) with a main effect of congruency (*F*(1,19) = 8.24, *p* = 0.010) (without significant interaction with the congruency).

We also tested the effect of congruency between 800 ms and 1300 ms, showing no significant LPC effect (main effect of congruency: *F*(1,19) = 0.21, *p* = 0.651) (all interactions with the congruency were non-significant, *p* > 0.05).

At DL3, we tested the effect of congruency to confirm the lack of ERP effect. Since grand-averaged ERPs did not suggest an *a priori* window of analysis for the N400 effect (or N2 effect) and the LPC effect, we used the same *a priori* windows than those at DL2 (*i.e.*, we assumed that the *a priori* windows at DL2 were the best reference).

Thus, we tested the effect of congruency between 250 ms and 800 ms, showing no significant N400 effect (main effect of congruency: *F*(1,19) = 0.69, *p* = 0.417) (all interactions with the congruency were non-significant, *p* > 0.05).

We also tested the effect of congruency between 800 ms and 1300 ms, showing no significant LPC effect (main effect of congruency: *F*(1,19) = 0.60, *p* = 0.449) (all interactions with the congruency were non significant, *p* > 0.05).

#### 3.2.3. Statistical Analysis of the Latency of the Congruency Effects

ERP effects were tested statistically with a repeated-measures ANOVA with semantic congruency (2 levels), anteroposterior (frontal, central, parietal), and laterality (left, midline, right) as within-participant factors and were computed for each DL using 50 ms windows (Table 4).

**Table 4:** Latency of the ERP Semantic Congruency Effects. C = Congruency; CA = Congruency × Anteroposterior interaction; CL = Congruency × Laterality interaction; CAL = Congruency × Anteroposterior × Laterality interaction; Statistical significance threshold: 0.01 (**) or 0.05 (*).

windows (ms)	DL0				DL1				DL2				DL3			
	C	CA	CL	CAL	C	CA	CL	CAL	C	CA	CL	CAL	C	CA	CL	CAL
0–50																
50–100																
100–150																
150–200	*				*		*									
200–250	**				*		**									
250–300	*				*		*									
300–350	**				**		*									
350–400	*				*				*							
400–450				*	**				*							
450–500			*	**	*											
500–550		*	**	*	**				*							
550–600	*	**	**	*					*							
600–650	**	**	*	*												
650–700	*	**	**						*							
700–750	**	**	**						*							
750–800	**	**	*		**											
800–850	**	*	**		*		**									
850–900	**	*	*		*	*	**									
900–950	**	*			**	**	*									
950–1000	**	**				**	**									
1000–1050	**					*	*									
1050–1100	*															
1100–1150	*					*										
1150–1200	*					*										
1200–1250						*										
1250–1300																
1300–1350																
1350–1450																
1450–1500																

At DL0, the effect of congruency corresponding to the N400 effect (and possibly N2 effect, see Discussion) was found between 150 ms and 400 ms. The effect of congruency (main effect of congruency and interactions with congruency) corresponding to the LPC effect was found between 400 ms and 1200 ms.

At DL1, the effect of congruency corresponding to the N400 effect (and possibly N2 effect, see Discussion) was found between 150 ms and 550 ms. The effect of congruency (main effect of congruency and interactions with congruency) corresponding to the LPC effect was found between 750 ms and 1250 ms.

At DL2, the effect of congruency corresponding to the N400 effect (and possibly N2 effect, see Discussion) was found between 350 ms and 750 ms. No other effect of congruency (main effect of congruency and interactions with congruency) was found.

At DL3, no effect of congruency (main effect of congruency and interactions with congruency) was found.

In summary, this analysis indicated that: (1) the N400 effect was delayed by increased degradation between DL0 and DL2 and (2) the LPC effect was delayed between DL0 and DL1.

## 4. Discussion

The aim of the study was to test the automaticity of the mechanisms underlying ERP effects elicited by final words in semantically incongruent sentences and final words in semantically congruent sentences. Sentences (including the final word) were presented with four levels of acoustic degradation (modulation with filtered or unfiltered pink noise, see the Methods Section). During the primary (ERP) study, participants performed a word recognition task (comparison between the target last word of the auditory sentence and a visual word displayed after the sentence presentation). Recognition performance was better when the visual word was semantically congruent with the sentential context under all levels of acoustic degradation indicating that the sentences were semantically processed even at the strong degradation level (DL3). A *degradation efficiency test* estimated (see the report in the Methods), with a semantic judgment task, whether sentential processing was impaired by acoustic degradation. The *degradation efficiency test* showed that participants were unable to discriminate incongruent from congruent sentences when sentences were strongly degraded (DL3), thus suggesting that if semantic processing remained at DL3, this mechanism was not controlled. The N400 effect and the LPC effect were not found at DL3. Delayed and attenuated ERP effects were found only under milder degradation (DL1 and DL2) where controlled sentence processing could not be ruled out according to the deviation from chance performance in the *degradation efficiency test* (see note [65]). These results suggest that both N400 and LPC effects recorded during the processing of a word within a sentential context likely reflect controlled rather than automatic mechanisms.

### 4.1. Behavioral Results

The performance of the primary study allows us to draw the following conclusions. (1) Recognition performance was affected by degradation, because RT and accuracy varied across DLs; (2) Participants recognized the target word at all DLs, at least at an automatic level (through a lexical/semantic and/or phonetic mechanism) as indicated by a significant target repetition effect even under the strongest degradation (DL3); (3) Sentences were semantically processed at all DLs because performance varied as a function of sentential semantic congruency in all conditions. The latter conclusion is in line with several studies showing semantic congruency effects in conditions of reduced controlled processing (e.g., [66,67,68,69,70,71]).

The comparison between the behavioral data of the primary study and the behavioral data of the *degradation efficiency test* yields to a further conclusion. While at DL0, DL1, and DL2, controlled and automatic sentence-level (and single-word priming) mechanisms may have influenced the processing of the target word, at DL3, the contribution of controlled-sentence level mechanisms were most likely negligible, leaving mostly automatic sentence-level (and single-word priming) processes. In the *degradation efficiency test*, participants were asked to perform a semantic congruency judgment on the same sentences and under the same DLs as presented in the primary study. At DL3, their discrimination performance was at chance level. Thus, controlled sentence-level mechanisms required to perform the task were unlikely to be activated at DL3. In summary, the behavioral data of the primary study suggest that sentences were processed at all levels of degradation including DL3 (Note: the behavioral data of the primary study taken alone did not indicate whether this sentential processing was automatic or controlled). The *degradation efficiency test* indicates that this sentential processing is likely to be automatic at DL3 and automatic and/or controlled at other degradation levels.

In the primary study, the recognition performance at DL3 was above chance while the performance on sentences semantic congruency judgment was at chance in the *degradation efficiency test*. This performance discrepancy can be explained by: (1) backward priming and/or (2) task differences.

During the primary study, the perception of the non-degraded visual words (presented after the sentences in order to perform the recognition task) may have influenced the perception of the target auditory final word of the sentence. This effect is known as “backward priming”. The non-degraded visual words may have reactivated the semantic representation of the degraded auditory target word to be recognized [13,72,73]. This effect would help to perform the recognition task but could not occur in the *degradation efficiency test*, wherein no visual words were presented. Although mainly reported for prime-target stimulus onset asynchrony (SOA) between 0 and 700 ms [33], backward priming has occasionally been found with larger SOAs [74,75]. We used a SOA larger than 1 s. Our data showed successful target recognition. This suggests that participants may have mentally rehearsed (at a phonological and/or semantic level) the degraded auditory sentence final word (*i.e.*, the target word) before the non-degraded visual word was displayed, in order to compare the representation of these two stimuli. Thus, although the time interval between these two stimuli was rather long, the time interval between the activation of their respective representations might still allow backward priming.

In addition to performance facilitation due to backward priming, the performance discrepancy between the primary study and the *degradation efficiency test* may arise from a difference in task difficulty. In the primary study, the task was a single-word recognition. In the *degradation efficiency test* the task was a sentential semantic judgment. The recognition task was expected to be much easier than the sentential semantic judgment task. Indeed, recognition of a single degraded word is easier than recognition of all (or most of) the words of a sentence at a given DL (*i.e.*, a necessary although not sufficient requirement for judging the semantic congruency of the sentence). Furthermore, these tasks may have engaged different mechanisms. Judging the semantic congruency likely requires complex words manipulations and syntax processing in order to process the whole sentence. Conversely, deciding whether a written visual word matches a previously presented auditory word may activate somewhat more elementary mechanisms such as word-based phonological/orthographic/lexical processes [76,77].

In summary, the performance discrepancy between the primary study and the *degradation efficiency test* was most probably due to backward priming, a task difference, or both effects combined. The *degradation efficiency test* indicated that sentence-level mechanisms that occurred at DL3 were most likely automatic. If sentence-level controlled mechanisms remained, the contribution of these was rather negligible. Thus, if electrophysiological (or behavioral) semantic processing effects were found at DL3, they would probably reflect automatic rather than controlled mechanisms.

The behavioral data of the primary study suggested that a negative repetition effect took place, because at all DLs including DL3, participants made more errors in the recognition task when the visual word and the target were identical than when they were different. This negative repetition effect could reflect a clearly different phonetic representation of the two to-be-compared words in the condition where the visual word and the target were different. Alternatively, a speed/accuracy trade-off (as indicated by the opposite direction of the RT and accuracy effects) might have taken place. Importantly, neither the trade-off nor the backward activation could contribute to the observed ERP effects because the neurophysiological data were recorded before the presentation of the visual word.

In summary, the behavioral data of the primary study and the *degradation efficiency test* indicated that participants processed the sentences semantically at all DLs and the *degradation efficiency test* indicates that at DL3 this sentential processing would be automatic rather than controlled.

### 4.2. ERP Effects Reflect Controlled Processing

When there was no degradation (DL0), the two expected ERP semantic congruency effects were found: a larger parietal negativity to incongruent than to congruent targets around 400 ms followed by a larger late (around 600 ms) parietal positive complex to incongruent than to congruent targets. Polarity, latency ranges and the typical scalp topography permit us to identify the former difference as the N400 effect (and possibly N2 effect, see Discussion below), and the latter, as the LPC effect. Most importantly, under the strongest degradation (DL3), that is, in a condition where the *degradation efficiency test* indicates that sentence-level mechanisms at work were mostly automatic, there was neither an N400 effect nor a LPC effect, not even a slight trend on the grand averages (Figure 3 and Figure 4).

An unlikely explanation for the lack of ERP effects at DL3 would be an overlap of the N400 effect with the LPC effect. This overlap obviously requires that both effects have: (1) the same size and (2) the same latency range. Since in our results the two effects, when reliables, were of approximately the same size, one can argue that the requirement (1) might be met. In contrast, requirement (2) is unlikely. Since the N400 is rather rarely observed after 600 ms post-stimulus [1], the overlap hypothesis would require that the increase of degradation from DL2 to DL3 induces the reappearance of a LPC effect (no significant LPC effect was found at DL2) with an unlikely early latency range.

Another unlikely alternative explanation for the disappearance of the N400 and LPC effects at DL3 would be that the acoustic degradation was strong enough to alter the speech signal to the extent that it is not perceivable as speech any more. This explanation is contradicted by the behavioral data of the primary study and the *degradation efficiency test*, showing better performance to congruent sentences than to incongruent sentences, and hence that sentences were processed at DL3.

Although we expected that degradation would primarily alter controlled rather than automatic mechanisms, degradation in speech signal quality can affect automatic processing stages as well. Indeed, automatic pre-attentive ERP components (e.g., P1, N1, P2), show changes in brain responses by noise masking overlaid on auditory stimuli [60,78]. Thus, at DL3, early ERP components, such as N1 and P2 were most probably present but attenuated to a level that could not be differentiated from the inherent background EEG noise. Importantly, the N1 and P2 were already hardly identifiable at lower degradation conditions. Thus, with attenuation due to our strong degradation at DL3, the remaining N1 and P2 are expected to have small amplitudes and most probably to be unidentifiable within the background EEG noise.

In summary, the lack of ERP effects at DL3 suggests that the N400 effect and the LPC effects were most likely governed by controlled sentence-level mechanisms. 

Hence, our data are well in agreement with the results of Hahne and Friederici [31], and Schön and Besson [32], suggesting that the N400 effect obtained with sentences reflects controlled rather than automatic mechanisms.

While, in theory, there may be a continuum between automatic and controlled processes [14,44,45,46,47,48,49], and although our experimental design with four degradation levels was in part based on this view, the pattern of the N400 effect was rather in agreement with a dichotomic perspective [22,43]. Indeed, this effect was rather stable among DL0, DL1, and DL2 and disappeared at DL3. This *all-or-none effect* might have been due to a large difference in the degradation level between DL2 and DL3 as suggested by the performance difference between these two levels in the *degradation efficiency test*. Future research with more subtle performance differences between several levels of stimulus degradation would be useful to further assess this *all-or-none effect* pattern.

### 4.3. Controlled Mechanisms

Although the study was not intended to test the large set of theoretical models of the N400 and the LPC, some speculative hypotheses may be formulated on the basis of the obtained data. Federmeier [79] mentioned in her review two types of controlled sentence-level semantic mechanisms: (1) predictive (expectation-based) processes and (2) integrative processes. Our data do not fit very well with the classical predictive models. These models presume a two-step process. First, during the presentation of a sentence, a set of predictions are generated. Second, when the final word is presented, lexical access is facilitated if this word is in the set of predictions. The apparent delay of the N400 effect under “low” and “medium” degradation, compared with the non-degraded condition, could only reflect a delay of the second mechanism, *i.e.*, the lexical access. This would imply that the mechanisms of lexical access are controlled, but this conclusion would contradict the broadly accepted opinion that lexical access is largely based on automatic mechanisms (e.g., [26,76,77]).

Instead our data fit better with an integrative mechanism (e.g., [79]) or a mechanism of preparation for future integration [80].

The results of the LPC effect can be explained by controlled mechanisms. The LPC effect was delayed and reduced with degradation. This result could reflect a “patching” or “repair” mechanism [41,42,81]. Patching would be present when the participants can actually identify the meaning of the sentences (e.g., [34]) and be absent when such meaning cannot be identified. The lack of LPC effect at DL2 and DL3 would indicate that the identification of the meaning of the presented words within sentences was completely lost.

At DL2, the grand-average ERPs suggest that the lack of a LPC effect did not seem to reflect the lack of LPC. Instead, it might reflect the development of a LPC to congruent targets across DL0, DL1, and DL2.

Finally, the delayed and reduced N400 and LPC effects across DL may be interpreted as reflecting more general mechanisms of feed-forward and feed-back processes [80]. Kotchoubey [80] proposed a general framework underlying ERP negative and positive components. As concerns speech comprehension, the N400 would be elicited by uncertainties arising from the content of a message, which would build a “model of possible content” and mobilize neuronal assemblies to scan for further information needed to test this model. The LPC would be elicited when key information is obtained to upgrade this model. Thus, these ERP components would reflect a cortical activity underlying the control of verbal behavior. This control may be delayed or attenuated with increasing speech degradation as suggested by the pattern of ERP effects across DLs observed here.

### 4.4. The N2 Component

The N400 peak in the DL0, DL1, and DL2 conditions was preceded by another smaller negative peak (Figure 3). Although our data do not validate the independence of these two components, this early peak brings to mind the studies of Connolly *et al.* [28], Connolly and Phillips [82], and Hagoort and Brown [83] who identified an early negative component around 200 ms that precedes the N400 during auditory sentence presentation. Connolly and Phillips [82] proposed that this earlier component might indicate a mismatch between the initial acoustic/phonological features of a word and the expectancy established by the context.

### 4.5. Attentional/Strategic Effects

Sentences were presented in blocks of decreasing DL order. The use of a blocked format, rather than a mix of varying levels of degradation within the same block of trials may introduce: (1) an attentional effect because participants would alter their attention (and their motivation) to the target when it is more easily identifiable [84]; (2) an effect of fatigue/reduced arousal; and (3) a learning effect. Our data show that attention, fatigue, and learning, though most probably varying across blocks had: (1) no effect on the data recorded at DL3, since it was the first block; (2) a rather weak effect on the data recorded at other DLs, since these effects could not explain the observed pattern of data across blocks and since a replication of these ERP patterns was done within a mixed design [85,86]. Indeed, if variation of attention or fatigue across blocks had an effect on our data, this effect would be best seen at DL0 because it was the last block, *i.e.*, when these block effects are expected to be the strongest. However, the behavioral data of the primary study and those of the *degradation efficiency test* indicated that performance improved across blocks. In addition, the N400 effect and the LPC effect had the expected amplitude and latency at the last block according to the literature [1]. Thus, behavioral and ERP data indicate that, although attention and fatigue most probably varied across blocks, their effects on the data recorded at DL0, DL1, and DL2 were rather negligible. Furthermore, a learning effect across DLs seems also to be marginal. Indeed, a learning effect would most probably be found at DL1 and DL0, *i.e.*, only after sentences were once processed at a controlled level (at DL2) and then repeated. According to the literature, such learning (repetition) might result in a habituation (*i.e.*, amplitude decrease) of the N400 [87] and hence a reduced N400 effect. However, the size of the N400 effect did not vary significantly among DL2, DL1, and DL0. Thus, learning across blocks, though possibly taking place, did not seem to play a major role in our data.

It might be stated that an alternative, mixed design would be free of such possible intervening effects. However, a mixed design would have, in turn, its own pitfalls. If four DLs are used in a between-subject design, a strong noise due to between-group variation would be introduced. Alternatively, in a within-subject design, using different sentences to test the same condition would introduce other sources of noise. Indeed, given the complexity of a sentence, sentences could not be matched with sufficient precision on all relevant parameters.

## 5. Conclusion

To summarize, our results together with those of Hahne and Friederici [31] and Schön and Besson [32] suggest that the N400 effect and the LPC effect recorded during the processing of a word within a sentential context reflect controlled rather than automatic mechanisms. Further experiments with more subtle performance differences between several levels of stimulus degradation would be useful to test the *all-or-none effect* pattern observed between DL2 and DL3 (*i.e.*, ERP effects are present at DL2 and absent at DL3).

## Appendix

**Table A1:** Example of the semantic sentences used to build our speech material.

Sentential Context	Congruent Target	Incongruent Target
Si tu vas jouer dehors, n’oublie pas ton	manteau	pommier
Ils ont visité la France pendant les	vacances	tapis
La maîtresse a recopié l’exercice sur le	tableau	tambour
Parce qu’il a désobéi, sa mère lui a donné une	fessée	maison
Norbert l’a aidée à laver la	vaisselle	montagne
Souvent, les personnes âgées perdent la	mémoire	casquette
Cela fait trois ans qu’Irène a quitté son	mari	tirroir
Tous les jours, Paul lit le	journal	marteau
